# Pancreatic metastasis from lung adenocarcinoma

**DOI:** 10.1002/jgf2.612

**Published:** 2023-03-08

**Authors:** Naoki Aoyama, Tetsuro Inokuma, Yuki Nakanishi, Akihisa Fukuda

**Affiliations:** ^1^ Department of Gastroenterology and Hepatology Kyoto University Graduate School of Medicine Kyoto Japan; ^2^ Department of Gastroenterology and Hepatology Kobe City Medical Center General Hospital Kobe Japan

**Keywords:** gastrointestinal medicine, internal medicine, respiratory disease

## Abstract

TTF‐1 is a highly useful marker to assist in differentiating between pulmonary and nonpulmonary, nonthyroid adenocarcinomas. Our case shows that TTF‐1 is highly useful in differentiating between pancreatic metastasis from lung adenocarcinoma and primary pancreatic cancer, especially when the clinical course and imaging findings are not helpful for differentiating them.

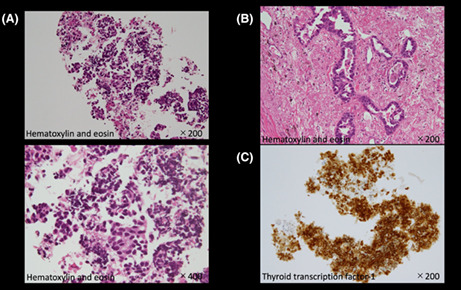

A man in his 70s underwent surgery for lung adenocarcinoma 5 years earlier, which recurred a year later. He undertook chemotherapy for multiple bone and lymph node metastases. Blood tests showed mild elevation of pancreatic enzymes (amylase 130 U/L, lipase 102 U/L) and CEA (75.2 ng/mL) during the course of time, but CA19‐9 was within the normal range (15.5 U/mL). A fluorodeoxyglucose‐positron emission tomography (FDG‐PET) showed fluorodeoxyglucose accumulation in the pancreatic head (Figure 1A). Contrast‐enhanced computed tomography (CE‐CT) detected a 7 mm hypovascular mass in the same region (Figure 1B). Endoscopic ultrasound (EUS) demonstrated an irregular hypoechoic mass with the dilation of the main pancreatic duct caudal to the mass (Figure 1C). EUS‐guided fine needle aspiration was performed for histological diagnosis. Hematoxylin and eosin (H&E) staining revealed adenocarcinoma, which resembled the histology of primary lung adenocarcinoma (Figure 2A,B). However, it was difficult to distinguish whether the pancreatic tumor was primary pancreatic cancer or metastatic tumor of lung cancer based on the findings of H&E staining alone. Immunohistochemistry showed positive expression for thyroid transcription factor‐1 (TTF‐1), a specific marker for the lung, leading to the diagnosis of pancreatic metastasis from lung adenocarcinoma (Figure 2C). As a result, we were able to select the appropriate treatment and he resumed chemotherapy for lung adenocarcinoma.

**FIGURE 1:**
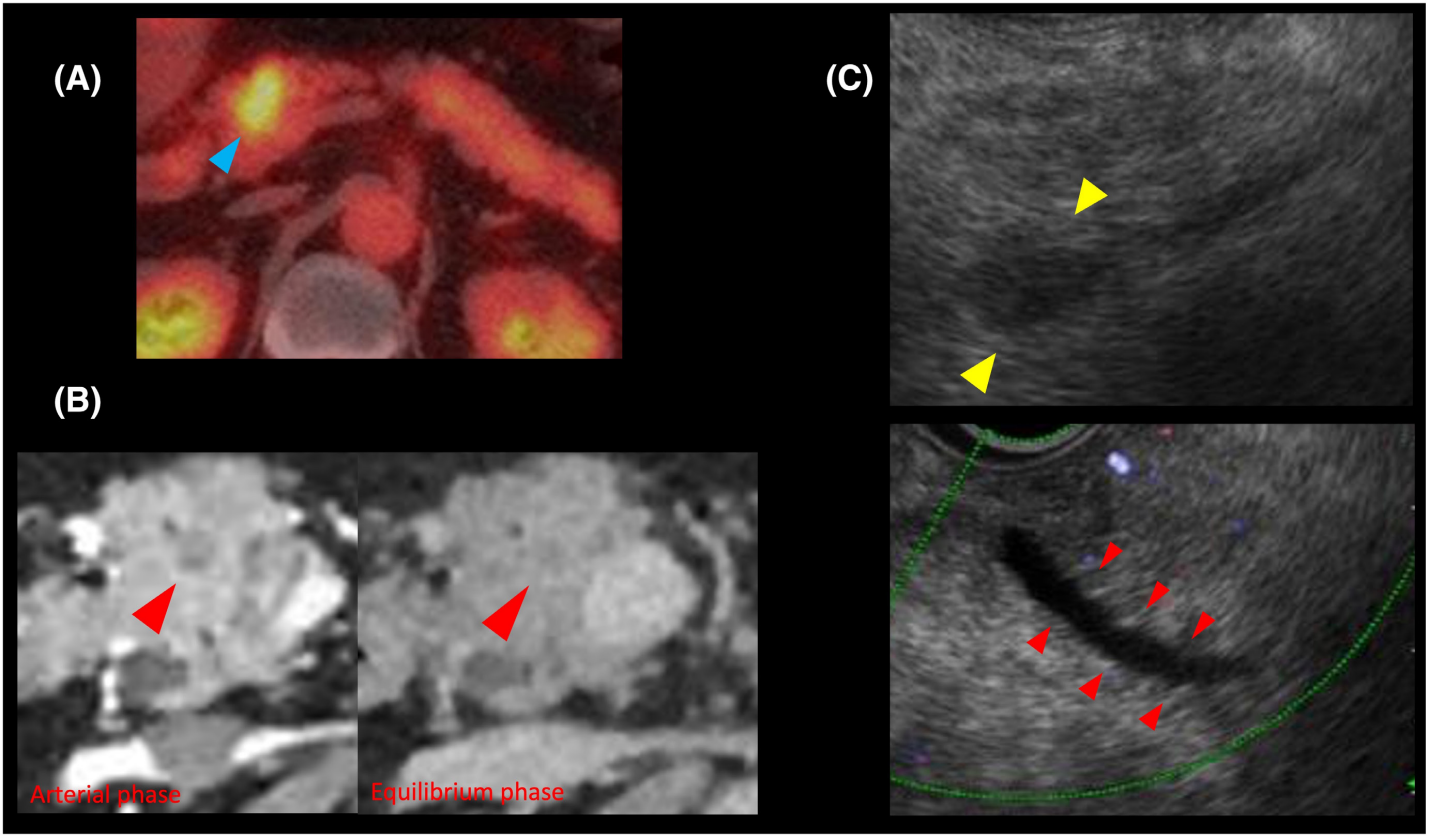
A fluorodeoxyglucose‐positron emission tomography (FDG‐PET) shows abnormal accumulation in the pancreatic head (A). Contrast‐enhanced computed tomography (CE‐CT) shows a 7 mm hypovascular mass (B). Endoscopic ultrasound (EUS) demonstrates an irregular hypoechoic mass with the dilation of the main pancreatic duct caudal to the mass (C).

**FIGURE 2:**
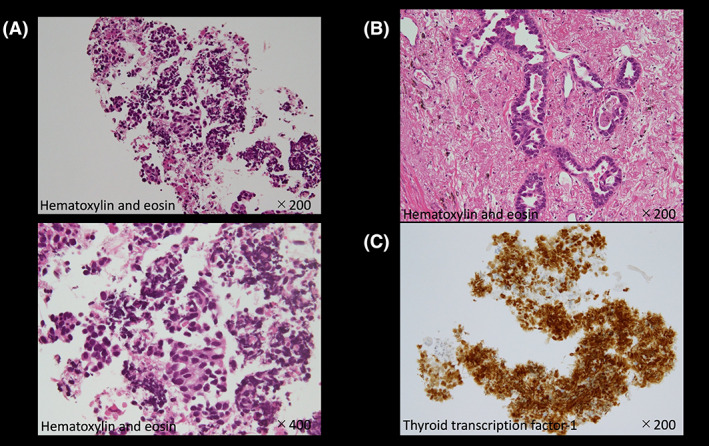
Hematoxylin and eosin (H&E) staining reveals adenocarcinoma (A), which resembled the histology of primary lung adenocarcinoma (B). Immunohistochemistry shows the positive expression for thyroid transcription factor‐1 (TTF‐1) (C).

Sweeney et al.^1^ reported that the most common primary cancer metastatic to the pancreas is kidney cancer (70.5%), followed by breast cancer (6.8%) and lung cancer (5.9%). TTF‐1 is a homeodomain‐containing tissue‐specific transcription factor, which is essential for the organogenesis and normal development of the thyroid, lung, ventral forebrain, and pituitary gland. When examined by organ, although the frequency varies among reports, TTF‐1 is reported to be positive in 76.7% of adenocarcinomas, 87.6% of small cell carcinomas, 4% of squamous cell carcinomas, and 39.6% of large cell carcinomas among lung cancers. On the contrary, TTF1 is positive in more than 90% of thyroid cancers. Napsin A, like TTF‐1, is a useful marker for lung adenocarcinoma and can be useful in differentiating lung cancer from thyroid cancer.^2^ In terms of histological type, there are few cases with positive TTF‐1 expression in adenocarcinomas derived from organs other than the thyroid gland and lungs, making it highly useful in distinguishing lung adenocarcinoma. TTF‐1 is highly positive in extrapulmonary small cell carcinomas (especially, in small cell carcinoma of the prostate, positive in 63.3%) and cannot be used to distinguish between lung primary and non‐lung primary in the case of small cell carcinomas.^3^

In conclusion, we report a case of pancreatic metastasis from lung adenocarcinoma. TTF‐1 is highly useful in differentiating between pancreatic metastasis from lung adenocarcinoma and primary pancreatic cancer, especially when the clinical course and imaging findings are not helpful for differentiating them.

## CONFLICT OF INTEREST STATEMENT

The authors have stated explicitly that there are no conflicts of interest in connection with this article.

## ETHICAL APPROVAL

Ethical approval was not required for this study.

## PATIENT CONSENT STATEMENT

Written informed consent was obtained from the patient for publication of this clinical image.

## CLINICAL TRIAL REGISTRATION

Not applicable.
